# Supplementary material for: Evolution of human leptospirosis in French Guiana, 2016–2022
Source: PLoS Negl Trop Dis. 2025 Oct 13;19(10):e0013620. doi: 10.1371/journal.pntd.0013620 (PMC12543287; doi:10.1371/journal.pntd.0013620)
Supplement: S1 Checklist — (DOCX) [file pntd.0013620.s001.docx]

STROBE Statement—Checklist of items that should be included in reports of ***cross-sectional studies***

# Item

|  | **No** | **Author’s formulation** |
| --- | --- | --- |
| **Title and abstract** | 1 | **(*a*) Not mentioned in the title, cross sectional design indicated in the abstract** |
|  |  | **(*b*) Informative and balanced summary of what was done**  **and what was found provided in the abstract and author summary** |
| **Introduction** |  |  |
| Background/rationale | 2 | **Sound scientific background and rationale indicated in the introduction** |
| Objectives | 3 | **Primary and secondary objectives indicated in the introduction** |
| **Methods** |  |  |
| Study design | 4 | **Study design described in the abstract and the first part of methods** |
| Setting | 5 | **Setting, location, time period and data collection methods described in the method section** |
| Participants | 6 | (*a*) **Eligibility criteria, sources and methods of selection of participants described in methods** |
| Variables | 7 | **Diagnostic criteria are clearly defined, each outcome and exposure is defined in methods** |
| Data sources/ measurement | 8* | **Data sources and measurements are detailed in methods** |
| Bias | 9 | **Efforts to address potential sources of bias are described in methods and discussed further** |
| Study size | 10 | **Study size described in results (no calculation of study size due to the nature of the study)** |
| Quantitative variables | 11 | **Described in methods and precised in the results** |
| Statistical methods | 12 | (*a*) **Statistical methods are described in the statistics part of methods** |
|  |  | (*b*) **Methods used to analyse subgroups are described in the statistical part of methods** |
|  |  | (*c*) **No adjustment for missing data** |
|  |  | (*d*) **Sampling accounted for by reporting number of cases on the estimated population of the year to permit comparison with the previous period** |
|  |  | (*e*) **Not applicable** |
| **Results** |  |  |
| Participants | 13* | (a) **Complete description of the selection process of participants detailed in figure 1** |
|  |  | (b) **Reasons for non participation given in figure 1 and result section** |
|  |  | (c) **Flow diagram as figure 1** |
| Descriptive data | 14* | (a) **Characteristics of study participants ( demographic, clinical, social) and**  **information on exposures and potential confounders are given in the results part** |
|  |  | (b) **Number of participants with missing data for each variable of interest is given in the tables** |
| Outcome data | 15* | **Main epidemiological characteristics and outcomes are summarized in tables** |
| Main results | 16 | (*a*) **Unadjusted estimates are given, no adjustment made** |
|  |  | (*b*) **Category boundaries when continuous variables were categorized are given** |
|  |  | (*c*) **Not applicable** |
| Other analyses | 17 | **Not applicable** |

**Discussion**

Key results 18 **Key results with reference to study objectives are given**



Limitations 19 **Limitations of the study, taking into account sources of potential bias or**

**imprecision, and direction and magnitude of any potential bias interpretation Interpretation** 20 **Results are interpreted considering objectives, limitations,**



**results from similar studies, and other relevant evidence** Generalisability 21 **Generalisability (external validity) of the study results is discussed**



# Other information

Funding 22 All fundings are disclosed

Checklist for reporting observational studies, adapted from the STROBE Statement (Strengthening the Reporting of Observational Studies in Epidemiology). Licensed under CC BY 4.0. Source: <https://www.strobe-statement.org/>; https://doi.org/10.1371/journal.pmed.0040296
