# Supplementary material for: Evolution of human leptospirosis in French Guiana, 2016–2022
Source: PLoS Negl Trop Dis. 2025 Oct 13;19(10):e0013620. doi: 10.1371/journal.pntd.0013620 (PMC12543287; doi:10.1371/journal.pntd.0013620)
Supplement: S1 Table — (DOCX) [file pntd.0013620.s002.docx]

**S1 Table. Collected variables**

| **Variable description** | **Type** |
| --- | --- |
| Year of birth | Date (aaaa) |
| Sex | 1 = Male  0 = Female |
| Country of birth | Qualitative variable among  1)France  2)French Guyana  3) Suriname  4) Brazil  5) Guyana  6) Haiti  7) Dominican Republic  8) other |
| District of residence | Qualitative among   1. Régina 2. Cayenne 3. Iracoubo 4. Kourou 5. Macouria 6. Mana 7. Matoury 8. St Georges 9. Remire Montjoly 10. Roura 11. St Laurent 12. Sinnamary 13. Montsinéry 14. Ouanary 15. Saul 16. Maripasoula 17. Camopi 18. Grand Santi 19. St Elie 20. Apatou 21. Awala yalimapo 22. Papaichton 23. Outside French Guyana |
| Referring medical center | 1)Cayenne  2) Kourou  3-) St Laurent du Maroni  4) Delocalized medical center |
| Year of arrival in French Guyana | Date aaaa |
| Comorbidity | Binary yes/no |
| Diabetes mellitus | Binary yes/no |
| High blood pressure | Binary yes/no |
| Obesity | Binary yes/no |
| Chronic kidney failure | Binary yes/no |
| Cardiopathy (all types) | Binary yes/no |
| Chronic respiratory failure | Binary yes/no |
| COPD | Binary yes/no |
| asthma | Binary yes/no |
| Sickle cell disease | Binary yes/no |
| Cancer | Binary yes/no |
| Hemopathy | Binary yes/no |
| HIV | Binary yes/no |
| Corticotherapy | Binary yes/no |
| Immunosuppressive treatment (other than corticosteroids) | Binary yes/no |
| cirrhosis | Binary yes/no |
| Pregnancy | Binary yes/no |
| Toxic substance use | Binary yes/no |
| Tobacco use at the time of hospitalization | Binary yes/no |
| Excessive alcohol consumption (WHO criteria) | Binary yes/no |
| Cannabis use | Binary yes/no |
| Crack cocaine use | Binary yes/no |
| Other toxic | Binary yes/no |
| **All exposures only notified if having occurred in the 21 days preceding the disease** |  |
| Research of exposure factor mentioned in the medical file (at least rodents and water) | Binary yes/no |
| Rodents exposure | Binary yes/no |
| Bovine exposure | Binary yes/no |
| Caprine exposure | Binary yes/no |
| Dog exposure | Binary yes/no |
| Cat exposure | Binary yes/no |
| Swine exposure | Binary yes/no |
| Exposure at home | Binary yes/no |
| Exposure outside of home |  |
| Gardening | Binary yes/no |
| Contact with urban freshwater at home | Binary yes/no |
| Contact with urban freshwater outside home | Binary yes/no |
| Contact with rural freshwater at home | Binary yes/no |
| Contact with rural freshwater outside of home | Binary yes/no |
| Regular exposure to rural water | Binary yes/no |
| Proximity with waste | Binary yes/no |
| Proximity with waste water | Binary yes/no |
| Flooding | Binary yes/no |
| Forest hiking | Binary yes/no |
| Living in informal settlement | Binary yes/no |
| Living in gold panning camp | Binary yes/no |
| Living in urban environment | Binary yes/no |
| Type of profession | Qualitative among   1. farmer 2. veterinarian 3. Building work 4. Sewer worker 5. fisherman (fresh water) 6. woodworker 7. butcher 8. gold mining 9. firefighter 10. « job » (informal work) 11. Working in an office 12. Retired 13. unemployed 14. cleaner 15. student 16. gardener 17. cook 18. other unexposed 19. other exposed |
| Health insurance | Qualitative among   1. social security 2. state medical aid (AME) 3. Universal complementary health coverage (CMUc) 4. No insurance, process ongoing 5. No rights, no paperwork done 6. Expired rights |
| Blood pressure (at admission) | Quantitative (mmHg) |
| Fever (T°>38° in the 12 hours following admission) | Binary yes/no |
| Oliguria (<500ml/24h) in the 48 hours following admission | Binary yes/no |
| Chills at home | Binary yes/no |
| Chills at admission | Binary yes/no |
| Asthenia (home or admission) | Binary yes/no |
| Anorexia (home or admission) | Binary yes/no |
| Headache at home | Binary yes/no |
| Headache at admission | Binary yes/no |
| Headache during hospitalization | Binary yes/no |
| Neck stiffness at admission | Binary yes/no |
| Neck stiffness during hospitalization | Binary yes/no |
| Seizure at home | Binary yes/no |
| Seizure at admission | Binary yes/no |
| Seizure during hospitalization | Binary yes/no |
| Cognitive impairment at home | Binary yes/no |
| Cognitive impairment at admission | Binary yes/no |
| Cognitive impairment during hospitalization | Binary yes/no |
| Conjunctivitis at home | Binary yes/no |
| Conjunctivitis at home | Binary yes/no |
| Conjunctivitis during hospitalization | Binary yes/no |
| Myalgia at home or at admission | Binary yes/no |
| Myalgias during hospitalization | Binary yes/no |
| Arthralgia at home or at admission | Binary yes/no |
| Arthralgia during hospitalization | Binary yes/no |
| Chest pain at home | Binary yes/no |
| Chest pain at admission | Binary yes/no |
| Chest pain during hospitalization | Binary yes/no |
| Dyspnea at home | Binary yes/no |
| Dyspnea at admission | Binary yes/no |
| Dyspnea during hospitalization | Binary yes/no |
| Cough at home | Binary yes/no |
| Cough at admission | Binary yes/no |
| Cough during hospitalization | Binary yes/no |
| Hemoptysis at home | Binary yes/no |
| Hemoptysis at admission | Binary yes/no |
| Hemoptysis during hospitalization | Binary yes/no |
| Diarrhea at home | Binary yes/no |
| Diarrhea at admission | Binary yes/no |
| Diarrhea during hospitalization | Binary yes/no |
| Nausea or vomiting at home | Binary yes/no |
| Nausea or vomiting at admission | Binary yes/no |
| Nausea or vomiting during hospitalization | Binary yes/no |
| Abnormal lung auscultation at admission | Binary yes/no |
| Abnormal lung auscultation during hospitalization | Binary yes/no |
| Abdominal pain at home | Binary yes/no |
| Abdominal pain at admission | Binary yes/no |
| Abdominal pain during hospitalization | Binary yes/no |
| Low back pain at home | Binary yes/no |
| Low back pain at admission | Binary yes/no |
| Low back pain during hospitalization | Binary yes/no |
| Abdominal guarding at admission | Binary yes/no |
| Abdominal guarding during hospitalization | Binary yes/no |
| Hepatomegaly during hospitalization | Binary yes/no |
| Splenomegaly during hospitalization | Binary yes/no |
| Purpura at admission | Binary yes/no |
| Purpura during hospitalization | Binary yes/no |
| Bleeding at home | Binary yes/no |
| Bleeding at admission | Binary yes/no |
| Bleeding during hospitalization | Binary yes/no |
| Jaundice at admission | Binary yes/no |
| Jaundice during hospitalization | Binary yes/no |
| Rash at home | Binary yes/no |
| Rash at admission | Binary yes/no |
| Rash during hospitalization | Binary yes/no |
| Suspicion of myocarditis (elevated troponins + compatible EKG anomaly or cardiac echography) | Binary yes/no |
| Leucocyturia (labstix or sediment) | Binary yes/no |
| Hematuria (labstix or sediment) | Binary yes/no |
| Proteinuria (labstix or urine biochemistry) | Binary yes/no |
| ASAT at admission | Quantitative (UI/l) |
| Bilirubin at admission | Quantitative (mmol/l) |
| TP | Quantitative (%) |
| Creatinine at admission | Quantitative (micromol/l) |
| Kaliemia at admission | Quantitative (mmol/l) |
| Natremia at admission | Quantitative (mmol/l) |
| Hemoglobin at admission | Quantitative (g/dl) |
| Leucocytes at admission | Quantitative (G/l) |
| Lymphocytes at admission | Quantitative (G/l) |
| Platelets at admission | Quantitative (G/l) |
| CRP at admission | Quantitative (mg/l) |
| ASAT maximal level | Quantitative UI/l |
| Date asat maximal level | Date dd/mm/aaaa |
| Bilirubin maximal level | Quantitative mmol/l |
| Date bilirubin maximal level | Date dd/mm/aaaa |
| Creatinine maximal level | Quantitative mg/l |
| Date creatinine maximal level | Date dd/mm/aaaa |
| Platelets minimum level | Quantitative G/l |
| Date Platelets minimum level | Date dd/mm/aaaa |
| Aspect of the CSF | Qualitative among   1. normal 2. meningitis with predominance of lymphocytes (>50%) 3. meningitis with predominance of neutrophils (>50%) 4. Not performed 5. meningitis, without formula (<20cells) 6. hemorrhagic |
| Date of symptoms onset | Date dd/mm/aaaa |
| Date of 1st medical contact | Date dd/mm/aaaa hh/mm |
| Date 1st diagnosis prescription (PCR, IgM ELISA or MAT) or 1st evocation in the medical chart (whichever happens first) | Date dd/mm/aaaa hh/mm |
| Medical specialty evoking diagnosis first | Qualitative among   1. general practitioner 2. emergency medicine 3. infectious diseases 4. reanimator 5. other |
| Date of 1st blood PCR | Date dd/mm/aaaa |
| Result 1st PCR | Binary positive/negative |
| Date of 2^nd^ blood PCR | Date dd/mm/aaaa |
| Result of 2^nd^ blood PCR | Binary positive/negative |
| Date of 1st urine PCR | Date dd/mm/aaaa |
| Result of 1^st^ urine PCR | Binary positive/negative |
| Date of 2^nd^ urine PCR | Date dd/mm/aaaa |
| Result of 2^nd^ PCR | Binary positive/negative |
| Result of CSF PCR | Binary positive/negative |
| Date of PCR in CSF | Date dd/mm/aaaa |
| Result 1st MAT | Binary positive/negative |
| Date 1st MAT | Date dd/mm/aaaa |
| Result 2^nd^ MAT | Binary positive/negative |
| Date 2^nd^ MAT | Date dd/mm/aaaa |
| Serogroup identified on the latest MAT realized | Qualitative among   1. Icterohemorrhagiae 2. Canicola 3. Copenhageni 4. Gryppotyphosa 5. Pyrogenes 6. Coagglutinin 7. other |
| Highest MAT level on the latest MAT | quantitative |
| MAT realized by the NRCL | Binary positive/negative |
| Date of the MAT realized by the NRCL | Date dd/mm/aaaa |
| Highest MAT level on the MAT realized by the NRCL | quantitative |
| Serogroup identified in the MAT realized by the NRCL | Qualitative among   1. Icterohemorrhagiae 2. Canicola 3. Copenhageni 4. Gryppotyphosa 5. Pyrogenes 6. Coagglutinin 7. Other |
| Result of 1st ELISA IgM | Binary positive/negative |
| Date of 1st ELISA | Date dd/mm/aaaa |
| Result of 2nd ELISA IgM | Binary positive/negative |
| Date of 2^nd^ ELISA | Date dd/mm/aaaa |
| Result of *lfb1* sequencing | Binary positive/negative |
| Serovar identified with *lfb1* | 1. Icterohemorrhagiae 2. Canicola 3. Copenhageni 4. Gryppotyphosa 5. Pyrogenes 6. other 7. not feasible |
| Species identified with *lfb1* | 1. Interrogans 2. Noguchi 3. other |
| Hospitalization (>24h stay) | Binary yes/no |
| Date of admission | Date dd/mm/aaaa |
| Date of discharge | Date dd/mm/aaaa |
| Antibiotherapy | Binary yes/no |
| Antibiotherapy with a drug active on leptospirosis | Binary yes/no |
| Date of antibiotic initiation | Date dd/mm/aaaa |
| Date of antibiotic discontinuation | Date dd/mm/aaaa |
| 3rd generation cephalosporin | Binary yes/no |
| doxycycline | Binary yes/no |
| penicillin | Binary yes/no |
| other antibiotic | Binary yes/no |
| Suspected paradoxical reaction | Binary yes/no |
| Sign of gravity (eg renal replacement therapy, vasopressor agent, mechanical ventilation, death) | Binary yes/no |
| Oxygen requirement | Binary yes/no |
| Date of Oxygen requirement | Date dd/mm/aaaa |
| Hypotension requiring fluid administration | Binary yes/no |
| Date of fluid administration | Date dd/mm/aaaa |
| Admission in intensive care unit | Binary yes/no |
| Date of admission in intensive care unit | Date dd/mm/aaaa |
| Administration of vasopressor agent | Binary yes/no |
| Date of administration of vasopressor agent | Date dd/mm/aaaa |
| Mechanical ventilation | Binary yes/no |
| Date of mechanical ventilation | Date dd/mm/aaaa |
| Renal replacement therapy | Binary yes/no |
| Date of renal replacement therapy | Date dd/mm/aaaa |
| Death | Binary yes/no |
| Date of death | Date dd/mm/aaaa |
