# Supplementary material for: Evolution of human leptospirosis in French Guiana, 2016–2022
Source: PLoS Negl Trop Dis. 2025 Oct 13;19(10):e0013620. doi: 10.1371/journal.pntd.0013620 (PMC12543287; doi:10.1371/journal.pntd.0013620)
Supplement: S2 Table — (DOCX) [file pntd.0013620.s003.docx]

**S2 Table. Biological characteristics of patients with leptospirosis in FG, 2016-2022 ***

|  | **Results** |
| --- | --- |
| **Blood** | **Median (25-75 IQR** |
| Platelet count, x1000/mm^3^ (N=178) | 127 (74-195) |
| Lowest platelet count, x1000/mm^3^ (N=167) | 91 (45-151) |
| Time to nadir of platelet count**, days (N=166) | 5 (4-7) |
| Creatinine mg/l (N=169) | 12.1 (9.2-21.3) |
| Maximal creatinine, mg/l (N=164) | 15.7 (10.8-33.0) |
| Time to maximal creatinine**, days (N=160) | 5 (4-7) |
| Bilirubin, µmol/L (N=171) | 13 (7-45) |
| Maximal bilirubin, µmol/L (N=163) | 25 (9-109) |
| Time to maximal bilirubin**, days (N=161) | 6 (3-9) |
| ASAT, IU/l (N=167) | 51 (28-95) |
| Maximal ASAT, IU/l (N=164) | 92.2 (47.4-157.5) |
| Time to maximal ASAT**, days (N=162) | 6 (4-9) |
| Prothrombin Time, % (N=138) | 79 (71-90) |
| CRP, mg/l (N=183) | 201 (81.2-286) |
| Hemoglobin, g/dl (N=179) | 12.9 (11.7-14.3) |
| Neutrophil count, x1000/mm^3^ (N=177) | 8.3 (5.8-11.5) |
| Lymphocyte count, x1000/mm^3^ (N=175) | 0.7 (0.5-1) |
| Natremia, mmol/l (N=174) | 133 (131-136) |
| Kalemia, mmol/l (N=162) | 3.5 (3.1-3.9) |
| **Urine** | N (%) |
| Proteinuria | 62/116 (53.5) |
| Hematuria | 78/144 (54.2) |
| Leukocyturia | 38/144 (33.6) |

IQR : interquartile; ASAT : aspartate aminotransferase; CRP : C-reactive protein

N is patients with available data, n/N is given for categorical data;

* biological characteristics are given on first obtained analysis unless specified

** since onset of symptoms.
