# Supplementary material for: Evolution of human leptospirosis in French Guiana, 2016–2022
Source: PLoS Negl Trop Dis. 2025 Oct 13;19(10):e0013620. doi: 10.1371/journal.pntd.0013620 (PMC12543287; doi:10.1371/journal.pntd.0013620)
Supplement: S3 Table — (DOCX) [file pntd.0013620.s004.docx]

**S3** **Table.** Details of biological diagnosis of the 188 study patients with leptospirosis

| **Test and timing of sampling since symptoms onset*** | **Tested patients** | **Patients with positive test** |
| --- | --- | --- |
| **Blood PCR**   - <5d - >5d | 137/188 (72.9)   - 82/136 (60.3) - 54/136 (39.7) | 96/137 (70.1)   - 71/82 (86.6) - 25/54 (46.3)* |
| **Urine PCR**   - <5d - >5d | 61/188 (32.4)   - 21/61 (34.4) - 40/61 (65.6) | 51/61 (83.6)   - 18/21 (85.7) - 33/40 (82.5) |
| **CSF PCR**   - <5d - >5d | 6/188 (3.2)   - 2/6 (33.3) - 4/6(66.7) | 2/6 (33.3)   - 0/2 (0) - 2/4 (50.0) |
| **ELISA IgM**   - <5d - >5d | 136/188 (72.3)   - 48/132 (36.4) - 84/132 (63.6) | 87/136 (64.0)   - 16/48 (33.3) - 68/84 (80.9) |
| **First MAT**   - < 7d - > 7d | 35/188 (18.6)   - 15/32 (46.9) - 17/32 (53.1) | 24/35 (68.6)   - 6/15(40.0) - 17/17 (100.0) |
| **Second MAT****   - <14d - >14d | 4/35 (11.4)   - 0/3 (0) - 3/3 (100)** | 3/4 (75.0)   - 0 (0) - 3/3 (100) |

Data are n/N (%) to indicate when missing data, N is patients with available data

CSF Cerebrospinal fluid, d days, IgM Immunoglobulin M, MAT Microscopic Agglutination Test

* Data were missing for timing analysis for: blood PCR (n=1), ELISA IgM (n=4), first MAT (n=3), second MAT (n=1)

** Only MAT performed in routine diagnosis and not ancillary analysis are presented here
