# Supplementary material for: Evolution of human leptospirosis in French Guiana, 2016–2022
Source: PLoS Negl Trop Dis. 2025 Oct 13;19(10):e0013620. doi: 10.1371/journal.pntd.0013620 (PMC12543287; doi:10.1371/journal.pntd.0013620)
Supplement: Attachment — Submitted filename: evolepto response to reviewers clean lining.docx [file pntd.0013620.s007.docx]

**Response to reviewers (in bold)**

The description of the diagnostic methods is fragmented and presented out of sequence, which makes it difficult for the reader to follow. For example, the microscopic agglutination test (MAT) is first mentioned on line 148 but only fully introduced on line 165. Similarly, diagnostic cut-off values for MAT are discussed before the leptospira serovars used in the assay are described. For clarity, each diagnostic method should be presented in a consistent order: define the test and its abbreviation at first mention, describe the methodology, outline cut-off values, and then explain the rationale for these parameters. Abbreviations should be introduced once and then used consistently throughout.

**We modified the different parts according to the reviewer’s suggestions to gain in clarity. We reorganized entirely the section now entitled “*Biological diagnosis, case definitions and inclusion criteria*”.**

Line 143-181

The rationale for the selection of leptospira serovars used in the MAT is not explained. Line 166 states that 11 antigens were used routinely in “Biomnis lab,” but it is unclear what this laboratory is and why these serovars were chosen. Was this decision informed by previous local isolation of invasive Leptospira strains, historical epidemiological data, or another factor? In addition, it is not clear whether serovars were tested individually or in pooled form. The description in lines 154–156 suggests individual testing in some cases, but this should be stated explicitly.

**The panel of antigens used by BIOMNIS laboratory for MAT analysis was their standard MAT panel which comprised 11 antigens. It was not specifically designed for French Guiana. Of note since 2022 this test is no longer performed at BIOMNIS laboratory but only at the NARCL. Concerning the study, using a panel that is possibly not designed for local studies was a limitation for sure because some infections caused by not tested serogroups might have been overlooked. Ho it was the only data available of routine MAT analysis during the study period. Of note BIOMNIS laboratory stopped performing the MAT by the end of 2022. Currently, only the national reference on leptospirosis performs this test. Of note, a broader panel, based on historical epidemiological data of French Guiana, was used for the ancillary MAT analysis at the national reference center for leptospirosis specifically on the 2021-2022 cases. Sera analyzed by MAT were tested separately against each serogroup of the antigenic panel.**

**L163-167**

***“******The microscopic agglutination test was performed in routine practice by BIOMNIS lab using a standard antigen panel not specifically designed for French Guiana. This panel contained 11 serovars: Icterohaemorrhagiae, Copenhageni, Canicola, Australis, Pyrogenes, Pomona, Autumnalis, Grippotyphosa, Castellonis, Sejroe and Patoc. Sera analyzed by MAT were tested individually against each strain of the antigenic panel.»***

Also, the diagnostic thresholds used for case classification require justification. The definitions of “certain” (≥1:400) and “probable” (1:200) cases given in lines 147–149 should be referenced to relevant guidelines or prior literature.

**The cut off of 1:400 for the definition of certain has already been suggested in previous studies (summarized in Valente et al doi:10.1186/s12879-023-08935-0). We added some references in the methodology section to support this choice (see Valente et al.**, **Wuthiekanun et al.** **10.3201/eid1301.060718 , Sukmark et al.)**

**See line 169-173**

***“For the study, the diagnosis of leptospirosis was confirmed if there was either a positive PCR in a biologic sample (blood, urine or cerebrospinal fluid) or a positive MAT titer of 1:400 or higher [3][14][15][16][17](except for Patoc) as previously reported. The diagnosis of leptospirosis was considered probable in the case of MAT positive titer of 1:200 (except for Patoc).”***

Similarly, the classification of probable cases based solely on anti-Leptospira IgM levels above 50 IU/ml (lines 149–152) without positive MAT or PCR requires explanation.

**We share the reviewer’s concern on the difficulty raised by using anti Leptospira IgM as probable case. On the one part, the classification of probable cases based solely on anti-leptospira IgM without MAT or PCR may be questionable. This is especially the case if differential diagnoses are proven in the patient because false positive have been reported. Ideally, all positive IgM should be confirmed with a MAT; which is not done in routine unfortunately. On the other part, using IgM as a biological marker of probable acute leptospirosis is commonly used in several studies (Adesiyun et al, 2011, Katz et al, 2008), and also implemented in some guidelines. For example, in 2025, the CDC confirmed its previous laboratory case definition based on positive IgM to retain a presumptive laboratory evidence (see https://ndc.services.cdc.gov/case-definitions/leptospirosis/). In our study, subjects with isolated IgM for diagnosis were eventually considered as probable cases only when no alternate diagnosis was proven and reported in the medical record. This supplementary criterion aimed to limit the inclusion of subjects with false positive IgM.**

**See line 173-177**

***“Patients with positive anti-Leptospira IgM above 50 IU/ml alone (without positive MAT or PCR) were considered as probable cases only if no differential diagnosis was found in the medical records, whether IgM seroconversion occurred or not. Patients with positive IgM levels alone (without MAT or PCR criteria) and with a*** ***proven alternate diagnosis were not included to limit the inclusion of subjects with false positive IgM.”***

Please confirm whether this was an ELISA and provide further detail on the assay itself.

**The detection of anti-Leptospira IgM was based on a standardized ELISA method. The mention of ELISA, as well as the details on the assay itself were provided in the methods section.**

**lines 156-160**

***Serological diagnosis of anti-Leptospira immunoglobulin M (IgM) was performed in laboratory Cerba or Biomnis using an enzyme-linked immunosorbent assay (ELISA) (Serion, Germany)[11][12]. Only positive tests were considered for this study. An equivocal test was considered as negative [13]. When the IgM titers were available in the laboratory database, only titers above a significant threshold of 50 IU/ml were defined as positive.***

The presentation of the PCR data contains several inconsistencies that need resolution. In the results (line 342), it is stated that PCR diagnosis was performed in 143 patients, but the flow chart in Figure 1 shows 1797 patients tested, with 117 confirmed cases by PCR. These figures appear contradictory and should be reconciled.

**We apologize for the confusion. The flowchart depicts all PCR tests performed during the study period that were screened to identify the eligible patients. Thus, it represents both the patients with positive and negative tests. This explains the high number of biological tests in the first line of flow chart. In the results section, except from the flow chart figure, only the data on the 188 patients included were presented. Among them, 143 benefitted from a PCR test (either on blood, urine or CSF sample) with 117 having at least one positive PCR test. Some details on the leptospirosis biological diagnosis tests were provided in a new table (S2) for the revised version of the manuscript (see below).**

Additionally, the Materials and Methods section should clearly describe the types of samples collected for PCR, the justification for using different sample types and the timing of collection in relation to illness onset. This is important, as timing can have a significant effect on positivity rates. In the results (lines 342–345), findings from different sample types are presented, but this information should be introduced earlier in the methods to provide context;

**We thank the reviewers for this valuable suggestion, some details on the different tests as well as their sampling timing are now provided as a supplementary table (table S3).**

**The justification for using different sample types was not ascertainable here with a retrospective scope. As added in the methods**

**Line 145-148**

***“The choice of diagnosis test for leptospirosis was based determined by the attending clinician. In particular, PCR testing could be requested by clinicians on the sample type of their choice, according to their clinical judgment, as no standardized protocol was in place within the hospitals during the study period.”***

Lines 346–347 suggest that acute and convalescent sampling was performed, with first MAT tests conducted in 51/188 patients and a second in only 7/51. If these represent acute and convalescent pairs, please provide details on the number of days from illness onset to the first sample, the interval between acute and convalescent collections

**To be more precise on this point we also added these results in the supplemental table S3.**

and any reasons for the low rate of paired sampling

**The low rate of paired sampling can be explained by local logistical constraints and by the change in the change in the conditions for reimbursement of the test by the French health insurance system. First, many patients of French Guiana are not seen to collect convalescent samples during follow up medical consultation because they have mobility difficulties to come to hospital (financial or transport) or because they feel better. And when the patient is seen in the convalescent period it is very rare that a MAT is performed in routine. Because it is not refunded by the French social health care anymore since 2014 (see Le Turnier et al. AJTMH 2014) laboratories don’t routinely transfer the sample for MAT analysis at the NRC of leptospirosis when IgM ELISA is positive. Finally, the prescription of MAT has been progressively abandoned since 2014 and very few practitioners continue to do it (in French Guiana as in the rest of France).**

As with the PCR data, ensure that these figures align with the flow chart in Figure 1.

**As explained above, the flow chart section concerned all patients screened for eligibility whereas the rest of the result section comprised only the data on included patients.**

Additional Considerations - It would strengthen the study to state whether in vitro isolation of Leptospira was attempted to confirm infecting serovars. This would provide an additional level of diagnostic confirmation and improve confidence in the epidemiological conclusions

**This a very relevant point. However, it was not performed during the study. So far, no Leptospira strain has ever been cultured from a French Guiana sample. Nevertheless, projects are currently conducted in French Guiana to isolate *Leptospira* strains from different samples: animal, human and environmental.**

Finally, the manuscript would benefit from careful language editing to improve clarity and readability, as well as to ensure that terminology is used consistently.

**The manuscript was entirely reviewed to improve syntax, clarity and readability.**

**Methods**

-Are the objectives of the study clearly articulated with a clear testable hypothesis stated?
-Is the study design appropriate to address the stated objectives?
-Is the population clearly described and appropriate for the hypothesis being tested?
-Is the sample size sufficient to ensure adequate power to address the hypothesis being tested?
-Were correct statistical analysis used to support conclusions?
-Are there concerns about ethical or regulatory requirements being met?

Reviewer #1: The study presents clear objectives aimed at updating the epidemiological and clinical characteristics of leptospirosis in French Guiana. While the hypothesis is not explicitly stated, the research questions and comparisons with previous findings suggest a well-defined investigative framework. The study design, incorporating multiple hospitals and a remote clinic, is appropriate for addressing these objectives, though variations in case identification methods between the two periods pose challenges for direct comparisons. Adjustments for population size may be necessary to ensure adequate statistical power for comparisons. Most statistical analyses appear robust; however, concerns arise regarding the use of raw case counts without adjusting for population differences. There are no concerns regarding ethical procedures.

**We thank the reviewer for this comment. The raw data of number of cases were provided for information purpose more than comparison. We acknowledge the reviewer’s concern regarding the potential bias that could be introduced by using these raw data for comparison between time periods. Hence, we removed the result of p value of comparison test between raw data. Now the comparison only appears between the 2007-2014 and 2016-2022 ratios of yearly mean number of cases reported to adult population in FG for each study year.**

Reviewer #2: okay

Reviewer #3: yes

**Results**

-Does the analysis presented match the analysis plan?
-Are the results clearly and completely presented?
-Are the figures (Tables, Images) of sufficient quality for clarity?

Reviewer #1: The analysis and results align with study aim. All tables need improvement.

**Tables were modified to gain in clarity and readability. We revised table 1 to separate sociodemographic characteristics from medical history as suggested, we also added a supplementary table describing the details of the diagnostic tests. We also removed the p-value comparing the raw cases in table 5 to avoid any misleading conclusion following a reviewer’s comment.**

Reviewer #2: okay

Reviewer #3: Yes

**Conclusions**

-Are the conclusions supported by the data presented?
-Are the limitations of analysis clearly described?
-Do the authors discuss how these data can be helpful to advance our understanding of the topic under study?
-Is public health relevance addressed?

Reviewer #1: The conclusion is shalow and the implications to public health is not properly addressed.

**The conclusion was modified to underscore some relevant public health findings.**

**“*The global burden of leptospirosis is increasing in French Guiana and the characteristics of the affected population is evolving. In the recent period, multiple exposures were frequent with domestic and non-domestic risks. As in other tropical areas, leptospirosis particularly affects vulnerable urban populations. Public health measures should integrate these findings to better target at-risk populations and provide locally adapted leptospirosis information and prevention campaigns*. »**

Reviewer #2: Results of the study are limited to the small study area in French Guiana
Otherwise okay

**Indeed, we agree with the reviewer on this point. The generalization of the finding s are limited to the area of French Guiana but provides useful insights into recent trends in the local epidemiology. However, we hope similar comparative studies could be performed in other settings to address the evolution of the local epidemiology.**

Reviewer #3: yes

**Summary and General Comments**

Reviewer #1: This study provides a comprehensive update on the epidemiological and clinical characteristics of leptospirosis in French Guiana. It is highly relevant, however, the presentation of results is somewhat disorganised, and the tables require significant improvements.

**We paid attention to the reviewers comment and substantially change the organization of methods (see the firs comment of the response letter) especially the section “*Biological diagnosis, case definitions and inclusion criteria*” and modified the tables.**

I have a major concern regarding the statistical robustness of the comparison between the 2007-2014 study and the current study. Alone, the results from the 2016-2022 study are extensive and valuable. The effort of comparing two comprehensive research studies in the same country is valid. However, direct comparisons between the two periods must be approached with caution due to differences in data sources. Although the authors claim to have used similar case definitions in both studies, the manner in which cases were obtained differs. The earlier study (2007–2014) included only two hospitals, while the current study expands to three hospitals and a remote clinic centre. Given these discrepancies, even the total number of cases should be adjusted based on population size to improve the statistical power of comparisons

**This a relevant comment. We can provide an answer for the concerns regarding the case of remote health centers and Kourou Hospital Center. During the first study from 2007-2014 some patients managed in remote health centers were actually included. Indeed, the hospital center of Cayenne was already the referral center for remote health centers at this time and performed their biological analysis. As a consequence, patients managed in remote health care centers with available data were included. They were not specifically described as managed by remote health centers at that time unfortunately. Regarding the center of Kourou, it is true that no data was available for this center at the time of data collection for the 2007-2014 period. However, considering the limited number of cases in the 2016-2022 period for Kourou (n=8) it seems improbable that this could explain the large increase of leptospirosis cases observed in the recent study. This point was added to the discussion section.**

**We agree with the remark on the need to adjust the comparison based on population size. This was already done in the manuscript (see table 5 with comparison between 2007-2014 and 2016-2022). Again, to avoid any confusion we removed the p-value of the comparison test of the number of cases raw data.**

When comparing the number of cases per year between the two studies, a Student's t-test could be appropriate if the data meet the assumptions of normality and equal variance. However, raw case counts alone do not account for population differences, meaning a direct comparison may be misleading

**The remark is relevant, we tested the normality of the distribution and the equality of variance to use the Student’s t test.**

**Shapiro wilk s test results: 2007-2014 group : p=0.427; 2016-2022 group : p=0.585**

**The two p values are greater than 0.05, indicating a normal distribution.**

**Levene's test was used to verify the equality of variances. p=0.0588**

**The p value is slightly greater than 0.05, which suggests that the variances of the two groups (2007-2014 and 2016-2022) can be considered equal.**

Reviewer #2: The authors present results of serological surveys in French Guiana. The work is well done, but is of little significance to anyone outside of the study area.
Minor concern: the use of "evolution" in the title is not appropriate, since there is no evidence that the disease evolved

**The term evolution was used to qualify the population impacted by leptospirosis. According to us, some changes occurred in leptospirosis epidemiology between the two time periods 2007-2014 and 2016-2022 as showed in table 5. Some changes were significant (burden of disease, comorbidities, birth country, occupation, rapidity for diagnosis suspicion) while some were only notable trends (gender, age). This was mentioned in the author summary.**

***“Leptospirosis is a bacterial disease transmitted from animals, especially rodents, that can cause serious illness or death. In French Guiana, many people live in vulnerable conditions, such as informal settlements, with exposure to flooding and diverse animal reservoirs, making it important to update our knowledge of who is affected and how the disease spreads. This study updates the characteristics of patients diagnosed with leptospirosis from 2016 to 2022 and compares them with those from 2007–2014 using a similar and robust case definition. The overall burden of the disease, including severe and non-severe cases, doubled over the study periods. Significant changes included higher comorbidities, more patients born in Haiti, differences in occupation, and faster clinical suspicion. A trend toward older age and a higher proportion of female patients were also observed. Patients often live in precarious conditions and have multiple sources of exposure both at home and elsewhere. The proportion of severe cases remained similar. These findings highlight subtle shifts in the epidemiology of leptospirosis in French Guiana and provide insights to support more targeted public health interventions and the identification of at-risk populations*.”**

Reviewer #3: This study presents an updated analysis of leptospirosis surveillance data in French Guiana over a seven-year period (2016–2022), with comparisons to a prior dataset from 2007–2014. The authors report shifts in the geographic distribution of cases, a modest increase in the median age of affected individuals, continued male predominance, and greater use of PCR diagnostics. Seasonal patterns remain, but occasional off-season clusters were detected. The manuscript addresses a neglected tropical disease in a high-incidence region, and the comparison with historical data adds value

1. The study compared data from 2016–2022 with 2007–2014. Were the case definitions, diagnostic methods, and inclusion criteria identical across periods?

**The definition of cases was overall similar. However, there were two minor inclusion criteria differences between the two time periods. First, there was a difference in IgM threshold for defining a positive test : >25 IU/ml in the 2007-2014 period and >50 IU/ml in the 2016-2022 period. This difference comes from the fact that the laboratories which performed the IgM ELISA test decided to modify the positivity threshold in 2018 to increase specificity (unpublished data, personal communication A Barbry, abstract communicated to a French “interdisciplinary meeting on anti-infective chemotherapy” RICAI 2025). Using a higher threshold for IgM positivity - a stricter criterion though- could have conducted to a bias toward less detection of cases in the more recent period. This could have contributed to limit the phenomenon of increasing burden observed during 2016-2022. However, those differences have been considered minor enough to still permit the comparison between time periods considering that most cases were based on PCR and not IgM.**

**Second, as the MAT results in the 2016-2022 period came from BIOMNIS laboratory and not from the national reference center for leptospirosis as in the 2007-2014 period, the panel of serovar tested was different from the 2007-2014.Thus, we did not emphasize the comparison of circulating serogroups between the two periods in this study.**

2. The study reports seasonal peaks during the rainy season. Has it been tested statistically, and did you consider other environmental variables such as flooding or river levels?
**The remark is relevant as pluviometry has already been associated to leptospirosis increase of cases in French Guiana as in many other countries. In fact, we assume that it may be the most shared characteristic in leptospirosis epidemiology worldwide (at least in tropical areas). Actually, a larger study on environmental and spatial factors associated to the occurrence of cases should start soon in French Guiana . Flooding and drainage water levels as well as other environmental factors will be analyzed in this future work.**

3. The study did not present any serovar distribution. Without the serovar information, is it possible to identify reservoirs and tailor interventions?

**It was debated to do so in this study. However, there are several risks of overinterpretation of presumptive serovars due to cross-reactions between serovars. Thus, in accordance with the National reference center for leptospirosis we preferred to present the serogroup results. Nevertheless, we assume that serogroups data whether derived from strain sequencing or MAT analysis could help determine the reservoir and tailor certain interventions as mentioned in the Discussion section. We also clearly recognize the limitations of serogroup identification carried out as part of this work.**

**Line 496-504**

***“However, these data should be interpreted very cautiously as only few serogroups were identified in our study as MAT has not been routinely performed since 2014. Also, the difference in panels and laboratories performing the MAT in routine versus in the ancillary analysis is a limit to our study, even if there was only one discordance found,*** ***which was explained by the timing of the sample. In addition, lfb1 sequencing results were obtained for a limited period of the study. Overall, this may have reduced the diversity of serogroups compared with the 2007-2014 period, when MAT data was available throughout the study period, and realized solely by the NRCL.”***

4. Lines 139 to 143
Inclusion criteria > 15 years at diagnosis, and exclusion criteria <18 years at the time of the data collection.
What was the minimum age of the patient to be included in the study?

**The minimum age of patients included in the study was 15 years at the time they contracted the disease and were treated at participating centres. The threshold of 18 years of age for data collection was set for regulatory reasons. Indeed, the procedure for obtaining participation would have required additional steps to include patients under the age of 18 at the time of data collection. To clarify, only the 15-year threshold was retained in the revised diagram, and the age at the time of data collection (inclusion) appeared only in the methodology section, under the exclusion criteria.**

5.Lines 157-159
PCR diagnosis was based on an in-house real-time PCR targeting the 16S RNA coding
gene and LipL32 gene. All PCR were performed by Biomnis laboratory.
Any reference for the PCR. Are the amplicons sequence confirmed.

**More informations on the PCR methods were added to the methods section as follows:**

**Line 149-155**

***“PCR diagnosis was based on an in-house real-time PCR targeting the 16S RNA coding gene and lipL32 gene. PCR was performed on 200 µL of plasma, serum, or whole blood using the MagNA Pure 96™ system. DNA extracts were amplified with primer pairs targeting 16S rRNA, lipL32, and an internal control (Phage M13), with probes labeled FAM/TAMRA or VIC/TAMRA. Reactions were run for 45 cycles on ABI7500 or QS5 thermal cyclers. For urine, 2 mL were centrifuged (12,000 rpm, 30 min), 0.5 mL with pellet was retained, and the same protocol applied. All routine PCR were performed by Biomnis laboratory.”***

**We also provided the reference papers of the methods**

**Smythe LD, Smith IL, Smith GA, Dohnt MF, Symonds ML, Barnett LJ, McKay DB. 2002. A quantitative PCR (TaqMan) assay for pathogenic Leptospira spp. BMC Infect Dis 2:13.**

**Levett PN, Morey RE, Galloway RL, Turner DE, Steigerwalt AG, Mayer LW. 2005. Detection of pathogenic leptospires by real-time quantitative PCR. Journal of Medical Microbiology 54:45–49.**

6. Line 258
One hundred fifty-five patients (82.5%) patients initially
consulted in CHC
Why majority of the cases in CHC ?

**The CHC is the main hospital center of French Guiana. It serves the largest area of catchment of French Guiana. It is therefore the center with the largest activity especially from the emergency department and is where most of the patients refer to. It is also where the delocalized medical center’s samples are sent, explaining its predominance in our study.**

7. Figure 2: Why there is a high incidence in 2021? Was this linked to any specific outbreak or environmental factors?

**The years 2020-2022, and 2021 in particular, were marked by intense rainy season as discussed in the text.**

**Lines 412-414**

**“*Historical rainfalls in 2020, an especially 2021 and 2022 may have likely contributed to an increase in the number of cases, given that half the cases in our study were reported in 2021 and 2022.”***

**This probably accounted for the high incidence in cases in this period. See below for a complementary explanation in response to a reviewer’s comment.**

8. Year 2020, during the era of covid, the cases were still same as year 2019 or 2022, despite of lockdowns and quarantines. So even occupational exposure, the cases did not go down, is it something to do with the living sanitation to present rodents exposure.

**As mentioned above, 2020 AND 2021 were marked by heavy and exceptional rainfall in the FG region. In addition, the lockdown resulting from the Covid pandemic forced people to stay at home or close to their homes. If we assume that most patients were infected through exposure in and around their homes, this could explain greater exposure to contaminated damp environments among people confined to their homes and account for higher levels of contamination during this period. However, an increase in contamination among people who continued to engage in high-risk professional activities is also a possibility. As we have not studied this particular point, it is not possible to comment further on this subject.**

**This was discussed here LINE 412-425**

***“Historical rainfalls in 2020, an especially 2021 and 2022 may have likely contributed to an increase in the number of cases, given that half the cases in our study were reported in 2021 and 2022. During these years the confinement probably resulted in the precarious population spending more time at home, in unsanitary and damp environment, as well as being more exposed to rodents at home. These factors could account for the increased number of cases in 2020, despite the lockdown. Another potential driving factor may be the increase in the number of people living in informal settlements in FG over the last decade [6], a well-known risk factor for leptospirosis [25]. In the present study, a third of the population with available data reported living in informal housing, which may be underestimate given the potential reluctance to disclose this information by the patient. Informal settlements may disrupt ecosystems due to human activity in forested areas, including within cities[26]. This can reduce biodiversity, which has been associated with an increased risk of leptospirosis transmission, as observed for other zoonotic diseases in French Guiana[27][28].”***

9. Line 359 * Six patients with MAT analysis also had samples analyzed with lfb1 sequencing. Was the results same for the serogroup

**This is precised lines 368-380**

*“****The results obtained from the NRCL ancillary analysis (genomic and serological) with the routine MAT data were aggregated to describe the serogroups. Overall, a serogroup identification was obtained for 43 subjects. The identified serogroups were Icterohaemorrhagiae for 33 patients (77%) (seven with serovar Copenhageni, 15 with serovar Icterohaemorrhagiae and 11 with lfb1 sequencing in favor of serogroup Icterohaemorrhagiae), Canicola for 7 patients (16%) and Pyrogenes, Ballum and Panama 1 patient per each (2%). Nine had coagglutinins, including two with lfb1 sequencing in favor of L. interrogans serogroup Icterohaemorrhagiae. lfb1 sequencing result was considered over MAT performed at NRCL in a discordant case. In this case, lfb1 gene sequences analysis was in favor of L. interrogans serogroup Icterohaemorrhagiae while an early MAT suggested Pomona. For this patient, a second MAT performed on a convalescent serum sample in routine eventually confirmed the serogroup Icterohaemorrhagiae. Table 4 shows the lfb1 sequencing results and the details of serogroups.”***

1. **Author Summary:**

Authors should provide a non-technical summary that highlights the study’s significance in the broader context of life sciences, making the findings accessible to both scientists and non-scientists.

The current summary lacks sufficient background information on leptospirosis globally and in French Guiana. It mainly repeats the abstract rather than situating the findings within a larger framework.

Consider incorporating the well-articulated rationale from the Introduction in a more simplified and engaging manner.

**We agree with the reviewer’s comment and made the suggested changes accordingly to improve the author summary.**

***“Leptospirosis is a bacterial disease transmitted from animals, especially rodents, that can cause serious illness or death. In French Guiana, many people live in vulnerable conditions, such as informal settlements, with exposure to flooding and diverse animal reservoirs, making it important to update our knowledge of who is affected and how the disease spreads. This study updates the characteristics of patients diagnosed with leptospirosis from 2016 to 2022 and compares them with those from 2007–2014 using a similar and robust case definition. The overall burden of the disease, including severe and non-severe cases, doubled over the study periods. Significant changes included higher comorbidities, more patients born in Haiti, differences in occupation, and faster clinical suspicion. A trend toward older age and a higher proportion of female patients were also observed. Patients often live in precarious conditions and have multiple sources of exposure both at home and elsewhere. The proportion of severe cases remained similar. These findings highlight subtle shifts in the epidemiology of leptospirosis in French Guiana and provide insights to support more targeted public health interventions and the identification of at-risk populations.”***

2. **Introduction:**

Lines 80–82: The Costa et al. study estimated global annual incidence, not incidence for a specific year. Stating “global incidence around 1,000,000 cases per year in 2015” is incorrect, as the estimate was based on data up to 2008.

Lines 82–84: Similar issue—annual incidence should be clarified rather than tied to a specific year.

Lines 96–97: Provide context for the reported figure of 18,500 cases—how does this compare to previous numbers?

**We fully agree and revised the manuscript accordingly.**

**Lines 81-83**

***Leptospirosis is a worldwide bacterial zoonotic disease, caused by pathogenic Leptospira spp., with around 1,000,000 cases per year and 58,000 deaths estimated in a study published in 2015 [1].***

**Lines 97-99**

***« By late 2022, it was estimated that around 18,500 people were living in informal settlements along French Guiana’s coastline. Moreover, an increase of 1.8% of informal settlement building has been reported between 2015 and 2019. »***

Lines 105–107: While multiple mammals are reservoirs for *Leptospira*, some studies suggest biodiversity loss may be linked to increased transmission risk. Consider referencing Derne et al. (2011) on pathogen regulation and leptospirosis.

**We added that reference in the discussion to comment on the risk of contracting the disease in informal settlements.**

**LINE 422-425**

***“Informal settlements may disrupt ecosystems due to human activity in forested areas, including within cities. This can reduce biodiversity, which has been associated with an increased risk of leptospirosis transmission, as observed for other zoonotic diseases in French Guiana.”***

**Some references were also added to discuss the impact of informal settlements on biodiversity loss and on the association between this phenomenon and other zoonotic diseases observed in French Guiana (10.1016/j.meegid.2021.104916 and 10.1186/s40068-024-00359-2 )**

3. **Methods:**

Are the three hospitals included in the study representative of all leptospirosis cases in the country?

**We understand the reviewer’s concern. As the three hospitals included in our study, as well as delocalized centers, constitute the totality of the medical centers in French Guiana, we believe this study is representative of all leptospirosis managed in hospital wards or consultation in French Guiana. The only cases eventually not included in our study would be the rare cases that were not addressed to a hospital or health care center. We assume this would be a very limited part due to the difficulty of obtaining a laboratory diagnosis outside the hospital and the low number of prescriptions coming from laboratories outside hospital (BIOMNIS Alexia Barbry personal data).**

In the ‘Data Collection’ heading, consider summarising all collected data in a well-structured table.

**Following the reviewer’s valuable suggestion, we** **have added a detailed table in the supplementary data (Table S1) section to provide the list of the variables collected.**

Lines 165–168: Move this paragraph earlier, immediately after the description of MAT (lines 154–156). Also, define MAT upon its first mention (line 148).

**Thank you for this observation. Following the reviewer’s suggestion, we paid attention to immediately define the acronym after its first mention.**

Lines 169–178: Consider whether using different diagnostic methods across samples may limit direct comparisons—this is worth revisiting in the results section.

**While we acknowledge the reviewer’s comment, we believe that the use of different diagnostic methods is not a major point of weakness in our study. The diagnosis of leptospirosis has been defined as certain or probable similarly to the classification established by organism such as WHO or CDC, and often utilized in research articles. As the diagnosis of leptospirosis can still be considered certain with various diagnosis method, we believe our cases remain comparable. The choice of tests performed as well as cut-off have been discussed in other questions.**

**We understand the reviewer’s concern concerning the use of different diagnosis methods. The part mentioned in this comment concerns the ancillary analysis, which results were pooled with the routine laboratory tests. We addressed the potential differences of results in the result section.**

**lines 368-380**

***“The results obtained from the NRCL ancillary analysis (genomic and serological) with the routine MAT data were aggregated to describe the serogroups. Overall, a serogroup identification was obtained for 43 subjects. The identified serogroups were Icterohaemorrhagiae for 33 patients (77%) (seven with serovar Copenhageni, 15 with serovar Icterohaemorrhagiae and 11 with lfb1 sequencing in favor of serogroup Icterohaemorrhagiae), Canicola for 7 patients (16%) and Pyrogenes, Ballum and Panama 1 patient per each (2%). Nine had coagglutinins, including two with lfb1 sequencing in favor of L. interrogans serogroup Icterohaemorrhagiae. lfb1 sequencing result was considered over MAT performed at NRCL in a discordant case. In this case, lfb1 gene sequences analysis was in favor of L. interrogans serogroup Icterohaemorrhagiae while an early MAT suggested Pomona. For this patient, a second MAT performed on a convalescent serum sample in routine eventually confirmed the serogroup Icterohaemorrhagiae. Table 4 shows the lfb1 sequencing results and the details of serogroups.”***

**While we acknowledge the difference in MAT panels performed in routine and in the ancillary analysis, we would like to insist on the fact that the serogroup analysis is not a major point in our study, and we took care to underline this point in the discussion of the revised manuscript as previously commented in this response to reviewer.**

**Lines 496-504**

**« *However, these data should be interpreted very cautiously as only few serogroups were identified in our study as MAT has not been routinely performed since 2014. Also, the difference in panels and laboratories performing the MAT in routine versus in the ancillary analysis is a limit to our study, even if there was only one discordance found******,*** ***which was explained by the timing of the sample. In addition, lfb1 sequencing results were obtained for a limited period of the study. Overall, this may have reduced the diversity of serogroups compared with the 2007-2014 period, when MAT data was available throughout the study period, and realized solely by the NRCL.* »**

Lines 192–196: Shift to a new paragraph, as the topic transitions from exposure and diagnosis to clinical manifestations.

**We have implemented the proposed changes.**

Lines 209–217: This section discusses data collection rather than statistical analysis—consider revising for clarity.

**We have revised the section as suggested.**

4. **Results:**

Figure 1: The Methods section defines the age threshold as 15, yet the figure suggests 18. Ensure consistency.

**As precised above, the threshold of 15 yo was for the patients minimal age required at the moment of the diagnosis and patient management. The threshold of 18 years old was for the age at data collection, which could differ from several years from diagnosis. We understand the confusion that it might cause, and removed the 18yo threshold from the flowchart.**

Lines 255–257: If the study’s cases do not represent the totality of reported cases in health departments, calculating incidence becomes problematic.

**We understand the reviewer’s concern. As the three hospitals included in our study, as well as delocalized centers, constitute the totality of the medical centers in French Guiana, we believe this study is representative of all leptospirosis managed in hospital wards or consultation in French Guiana. The only cases eventually not included in our study would be the rare cases that were not addressed to an hospital or health care center. We assume this would be a very limited part due to the difficulty of obtaining a laboratory diagnosis outside the hospital and the low number of prescription coming from laboratories outside hospital (BIOMNIS laboratory, Alexia Barbry personal data). To avoid any mislead, we chose not to mention incidence in our article but rather to refer to the total number of cases per year. We however, to the best of our capacity, provided an exhaustive report of the cases in the participating centers, based on our diagnosis criteria.**

Tables and text should follow a logical sequence, separating socioeconomic characteristics from medical history (e.g., comorbidities, addictions). Align Table 1 accordingly—health insurance access belongs under socioeconomic status, not medical history

**Following the reviewer’s suggestion, we reformed the sections in table 1.**

Table 1: Specify the figures in parentheses—are they interquartile ranges (IQR), minimum/maximum values, or something else?

**The informations were provided in the legend figuring at the bottom of each table.**

**Ex table 1 “*Data are mean (standard deviation) or median (IQR) for quantitative variables and n (%) for categorical variables. In case of missing data, n/N is specified for categorical variables and N is provided for quantitative variables* »).**

Table 1: Each characteristic has a different sample size. If reporting with missing data, add a row explicitly stating missing values for each variable, rather than calculating percentages inconsistently.

Table 1: If the parentheses contain percentages, ensure this is clearly indicated.

**We thank the reviewer for this comment. As mentioned in the legend, in case of missing data, n/N is specified for categorical variables and N is provided for quantitative variables.**

**Moreover, we modified the methods section to precise how missing data were reported throughout the manuscript as follows:**

**Line 227-230**

**“*Quantitative variables were described as mean and standard deviation or median and interquartile range, depending on the variable distribution, and qualitative variables were described with frequency and percentage*** ***with n/N to indicate the number of available data for each categorical variable with missing data.”***

Separate exposure factors from sociodemographic characteristics for clarity.

**We have made the changes accordingly.**

Lines 290–292: Exposure descriptions are unclear due to mixing exposure types and exposure sites. Consider restructuring.

**In light of the reviewer’s comment, we restructured this section as follows.**

**Lines 297-300**

**“*The main reported exposure factors were the proximity with rodents and freshwater contact followed by forest hiking, living in informal settlements, and proximity with non-rodent mammals (Table 2). Overall, most patients in whom several exposure factors were investigated (84/138, 60.9%) had at least two reported exposure factors.***

***Concerning the exposure site, forty-four patients (65.7%) reported both domestic and non-domestic exposures (without accounting occupational exposure).* »**

**We noted that a mistake occurred when giving the details of domestic and non-domestic exposures in Table 2. The denominator used for each variable was 188 which was incorrect and led to provide wrong percentages. We corrected throughout the correct data including in the abstract.**

**We chose to reformulate each exposure site and modified the results as follows in table 2:**

| **Site of exposure** | |  | |  |
| --- | --- | --- | --- | --- |
|  | ≥ 1 domestic exposure | | 89/102 (87.3) | |
|  | ≥ 1 non-domestic exposure | | 76/87 (87.4) | |
|  | Both domestic and non-domestic | | 44/67 (65.7) | |
|  | Residence in urban area | | 149/171 (87.1) | |

Table 2: The denominator for “Non-occupational exposure” varies—explain why.

**We apologize for the confusion. Each non-occupational exposure was collected in medical files. The number of missing data varies for each exposure, as some exposures were less researched than others during routine care by physician, which is a limit of this retrospective study.**

Table 3: Specify the timing of follow-up assessments.

**Unfortunately, the date of the symptom apparition was not recorded during data collection. Thus, only the data « after admission » is available and could be given in the study results.**

Line 310–311: Consider rewriting as: “Among patients with bleeding signs (N=29), 10 (34.9%) ...” for improved readability.

The section’s flow would improve by placing lines 312–315 at the start, followed by lines 308–311, then Table 3, and finally lines 317–321.

Lines 315–316: Remove “Finally” and begin a new paragraph with “The median duration between onset of symptoms and…

**We have implemented the proposed changes for all of the three points listed above by the reviewer.**

**Lines 318-323**

**“*****The median duration between onset of the symptoms and first medical contact was 3.7 days (2.5-5.6, range 0-15). Regarding clinical management, leptospirosis was suspected upon hospital admission for 101 patients (53.7%). Median time between first medical contact and diagnosis suspicion was 12.2 (5.1-44.7) hours. Among patients with bleeding signs (N=29), 10 (34.9%) were categorized as having severe leptospirosis and 5/29 (17.2%) underwent mechanical ventilation.***

***Lines 333-334***

***“The median duration between onset of symptoms and antibiotic therapy initiation was 4 days (3-6).* “ was moved to the paragraph about antibiotic therapy.**

Supporting Table 2: Why is this table placed mid-manuscript rather than in supplementary materials? Ensure logical placement.

**We apologize for the error and placed table “Biological characteristics and diagnosis of the study population” in supplementary materials.**

The Laboratory results section is disorganised.

The number of PCR tests performed is mentioned, yet their results are absent.

**We thank the reviewer for pointing this out. Accordingly, we have revised the manuscript to include more information about the PCR tests**

**lines 349-353**

**“*A PCR test was performed for 143 (76.1%) of 188 study patients. Among them, the most frequent sample was blood for 137 patients (95.8%), positive for 96 cases, followed by urine for 61 patients (42.7%), positive for 51 cases, and cerebrospinal fluid for 6 patients (4.2%), positive for 2 cases. Both blood and urine PCR assays were performed in 55 patients (38.5%).* »)**

Similarly, MAT results lack clarity—specify titres.

**In light of the reviewer’s comment, we added this data to the manuscript**

**lines 355-366**

**“*As routine diagnosis, 35/188 (18.6%) patients were tested with MAT. The median delay between symptoms onset and first MAT was 9.5 [ 28-52 ] days; 24/35 (68.6%) MAT were positive. A second test was performed for 4/35 patients, after 7, 19 and 20 days since first MAT sampling (1 missing data). Three patients were already MAT positive and one seroconverted from a negative MAT to a titer of 1:3200. Overall, among the 25 positive MAT , the median titer was 1:1600 [1:800-1:3200].***

***The ancillary study provided complementary MAT results for 19 patients. Two of them already had a MAT in routine care. One seroconverted from 0 to a titer of 1:12800 after 20 days. The second had a positive MAT on both tests but a distinct presumptive serogroup : Pomona on the 1st MAT 1:400 and Icterohaemorrhagiae on the second MAT with titer 1:800. The details of the leptospirosis biological diagnosis of all the patients included is provided in supplementary table S3.*”**

Serogroup Identification: Were all 43 subjects analysed only through NRCL ancillary testing, or was MAT included?

**We aggregated the NRCL and routine MAT data to report the serogroups of the 43 subjects. To prevent misunderstanding, we have revised the text to clarify our argument (see a previous response of ours). Moreover, the detail of diagnosis tests has been added as a supplementary table (table S3 Details of leptospirosis biological diagnosis of the 188 study patients).Finally, during the revision process we identified an error in the reported number of MAT done in routine; we corrected it in the revised manuscript.**

Consider adding a supplementary table detailing results by panel.

**We carefully considered the reviewer’s recommendation, but opted not to implement it, as it would have only concerned a limited number of patients and didn’t appear that relevant for this paper. However, this could be proposed if the editor thinks it is a major point.**

Line 366: Clarify the meaning of numbers in parentheses

**The number in parentheses represents the standard deviation as precised in the line above *“****Compared to the 2007-2014 period, the mean (SD) annual number of included cases reported »****.***

5. **Discussion:**

Lines 381–382: Avoid repeating the study’s aim here—it is unnecessary.

**We concur with the reviewer’s suggestion and have modified the text accordingly.**

6. **Conclusion:**

Expand discussion on public health implications—what do these findings mean in practice?

**In light of the reviewer’s comment, we have elaborated on this aspect in the revised manuscript and largely modified the conclusion as follows:**

**“*The global burden of leptospirosis is increasing in French Guiana and the characteristics of the affected population is evolving. In the recent period, multiple exposures were frequent with domestic and non-domestic risks. As in other tropical areas, leptospirosis particularly affects vulnerable urban populations. Public health measures should integrate these findings to better target at-risk populations and provide locally adapted leptospirosis information and prevention campaigns*. »**
