# Supplementary material for: Evolution of human leptospirosis in French Guiana, 2016–2022
Source: PLoS Negl Trop Dis. 2025 Oct 13;19(10):e0013620. doi: 10.1371/journal.pntd.0013620 (PMC12543287; doi:10.1371/journal.pntd.0013620)
Supplement: Attachment — Submitted filename: Reviewer comments.docx [file pntd.0013620.s005.docx]

This study provides a comprehensive update on the epidemiological and clinical characteristics of leptospirosis in French Guiana. It is highly relevant, however, the presentation of results is somewhat disorganised, and the tables require significant improvements.

I have a major concern regarding the statistical robustness of the comparison between the 2007-2014 study and the current study. Alone, the results from the 2016-2022 study are extensive and valuable. The effort of comparing two comprehensive research studies in the same country is valid. However, direct comparisons between the two periods must be approached with caution due to differences in data sources. Although the authors claim to have used similar case definitions in both studies, the manner in which cases were obtained differs. The earlier study (2007–2014) included only two hospitals, while the current study expands to three hospitals and a remote clinic centre. Given these discrepancies, even the total number of cases should be adjusted based on population size to improve the statistical power of comparisons.

When comparing the number of cases per year between the two studies, a Student's t-test could be appropriate if the data meet the assumptions of normality and equal variance. However, raw case counts alone do not account for population differences, meaning a direct comparison may be misleading.

**Point-by-Point Comments:**

Abstract:

No comments.

Author Summary:

Authors should provide a non-technical summary that highlights the study’s significance in the broader context of life sciences, making the findings accessible to both scientists and non-scientists.

The current summary lacks sufficient background information on leptospirosis globally and in French Guiana. It mainly repeats the abstract rather than situating the findings within a larger framework.

Consider incorporating the well-articulated rationale from the Introduction in a more simplified and engaging manner.

Introduction:

Lines 80–82: The Costa et al. study estimated global annual incidence, not incidence for a specific year. Stating “global incidence around 1,000,000 cases per year in 2015” is incorrect, as the estimate was based on data up to 2008.

Lines 82–84: Similar issue—annual incidence should be clarified rather than tied to a specific year.

Lines 96–97: Provide context for the reported figure of 18,500 cases—how does this compare to previous numbers?

Lines 105–107: While multiple mammals are reservoirs for *Leptospira*, some studies suggest biodiversity loss may be linked to increased transmission risk. Consider referencing Derne et al. (2011) on pathogen regulation and leptospirosis.

Methods:

Are the three hospitals included in the study representative of all leptospirosis cases in the country?

In the ‘Data Collection’ heading, consider summarising all collected data in a well-structured table.

Lines 165–168: Move this paragraph earlier, immediately after the description of MAT (lines 154–156). Also, define MAT upon its first mention (line 148).

Lines 169–178: Consider whether using different diagnostic methods across samples may limit direct comparisons—this is worth revisiting in the results section.

Lines 192–196: Shift to a new paragraph, as the topic transitions from exposure and diagnosis to clinical manifestations.

Lines 209–217: This section discusses data collection rather than statistical analysis—consider revising for clarity.

Results:

Figure 1: The Methods section defines the age threshold as 15, yet the figure suggests 18. Ensure consistency.

Lines 255–257: If the study’s cases do not represent the totality of reported cases in health departments, calculating incidence becomes problematic.

Tables and text should follow a logical sequence, separating socioeconomic characteristics from medical history (e.g., comorbidities, addictions). Align Table 1 accordingly—health insurance access belongs under socioeconomic status, not medical history.

Table 1: Specify the figures in parentheses—are they interquartile ranges (IQR), minimum/maximum values, or something else?

Table 1: Each characteristic has a different sample size. If reporting with missing data, add a row explicitly stating missing values for each variable, rather than calculating percentages inconsistently.

Table 1: If the parentheses contain percentages, ensure this is clearly indicated.

Separate exposure factors from sociodemographic characteristics for clarity.

Lines 290–292: Exposure descriptions are unclear due to mixing exposure types and exposure sites. Consider restructuring.

Table 2: The denominator for “Non-occupational exposure” varies—explain why.

Table 3: Specify the timing of follow-up assessments.

Line 310–311: Consider rewriting as: “Among patients with bleeding signs (N=29), 10 (34.9%) ...” for improved readability.

The section’s flow would improve by placing lines 312–315 at the start, followed by lines 308–311, then Table 3, and finally lines 317–321.

Lines 315–316: Remove “Finally” and begin a new paragraph with “The median duration between onset of symptoms and ...”.

Supporting Table 2: Why is this table placed mid-manuscript rather than in supplementary materials? Ensure logical placement.

The Laboratory results section is disorganised.

The number of PCR tests performed is mentioned, yet their results are absent. Similarly, MAT results lack clarity—specify titres.

Serogroup Identification: Were all 43 subjects analysed only through NRCL ancillary testing, or was MAT included? Consider adding a supplementary table detailing results by panel.

Line 366: Clarify the meaning of numbers in parentheses.

Discussion:

Lines 381–382: Avoid repeating the study’s aim here—it is unnecessary.

Overall, this section is well-structured.

Conclusion:

Expand discussion on public health implications—what do these findings mean in practice?
